# Nanoimprint lithography steppers for volume fabrication of leading-edge semiconductor integrated circuits

**DOI:** 10.1038/micronano.2017.75

**Published:** 2017-09-25

**Authors:** S.V. Sreenivasan

**Affiliations:** 1 Department of Mechanical Engineering, NASCENT Center, The University of Texas at Austin, J.J. Pickle Research Campus, Building 160, 10100 Burnet Road, Austin, TX 78712, USA; 2 Canon Nanotechnologies Inc., Austin, TX 78758, USA

**Keywords:** jet and flash imprint, nanoscale overlay, nanoimprint defectivity, semiconductor fabrication, steppers, precision systems, UV nanoimprint

## Abstract

This article discusses the transition of a form of nanoimprint lithography technology, known as Jet and Flash Imprint Lithography (J-FIL), from research to a commercial fabrication infrastructure for leading-edge semiconductor integrated circuits (ICs). Leading-edge semiconductor lithography has some of the most aggressive technology requirements, and has been a key driver in the 50-year history of semiconductor scaling. Introducing a new, disruptive capability into this arena is therefore a case study in a “high-risk-high-reward” opportunity. This article first discusses relevant literature in nanopatterning including advanced lithography options that have been explored by the IC fabrication industry, novel research ideas being explored, and literature in nanoimprint lithography. The article then focuses on the J-FIL process, and the interdisciplinary nature of risk, involving nanoscale precision systems, mechanics, materials, material delivery systems, contamination control, and process engineering. Next, the article discusses the strategic decisions that were made in the early phases of the project including: (i) choosing a step and repeat process approach; (ii) identifying the first target IC market for J-FIL; (iii) defining the product scope and the appropriate collaborations to share the risk-reward landscape; and (iv) properly leveraging existing infrastructure, including minimizing disruption to the widely accepted practices in photolithography. Finally, the paper discusses the commercial J-FIL stepper system and associated infrastructure, and the resulting advances in the key lithographic process metrics such as critical dimension control, overlay, throughput, process defects, and electrical yield over the past 5 years. This article concludes with the current state of the art in J-FIL technology for IC fabrication, including description of the high volume manufacturing stepper tools created for advanced memory manufacturing.

## Introduction

In this section, the manufacturing requirements associated with advanced semiconductor lithography are discussed. This is followed by a discussion of lithographic approaches being used or being considered as future candidates for semiconductor fabrication. The section then discusses technology gaps in IC fabrication that establish the motivation for Jet and Flash Imprint Lithography (J-FIL) as a viable option for IC fabrication. J-FIL (referred to as S-FIL in early papers) stepper technology was conceived at the University of Texas at Austin, and then developed for semiconductor fabrication at Molecular Imprints Inc., a UT-Austin spin out; and is now part of Canon Corporation.

### Semiconductor lithography requirements

While several nanopatterning techniques are reported in the literature, only a small percentage of them have the potential to be viable in volume manufacturing of semiconductor ICs. Manufacturing viability requires that the patterning approach possess the following attributes: (i) high-resolution and tight pitch structures with long-range order; (ii) ability to simultaneously pattern different types of structures with varying pattern densities; (iii) very low pattern-placement distortions relative to an ideal grid; (iv) ability to overlay a pattern relative to a previous pattern with overlay errors of <1/4th the most aggressive pattern half-pitch; (v) low overall process defectivity to enable high IC yield; and (vi) high-throughput and high mask usage or template life to achieve acceptable cost of ownership. (In this article the term “template” is used to define the nanoimprint master and is synonymous with the term “mold” or the term “imprint mask” that are also used in the nanoimprint lithography literature.) Table 1 provides representative lithographic requirements for leading-edge memory devices^1^. Here, memory devices are used to define representative requirements, rather than logic chips, as memory devices have more aggressive and well-defined half-pitch requirements, whereas logic-device nodes are not clearly defined in recent years as discussed in Table 1 (Ref. 1). This table of specifications illustrates the daunting challenge a new lithographic technology faces when it is being considered as a manufacturing option for advanced semiconductor ICs. Optical projection lithography has been the workhorse in this industry for 50 years^2^, and has benefited from years of sustained research-and-development investments from industry and academia. In its current form, 193 nm wavelength immersion (193i) photolithography (PL) has incrementally incorporated numerous scientific innovations (for example, excimer lasers, precision fabrication of large-scale optics, magnetically levitated stages with nanoscale accuracy, chemically amplified photoresists, to name a few). Even though diffraction limits its ultimate resolution to ~80 nm minimum pitch for lines and spaces and ~110 nm pitch for more complicated pattern, replacing PL with a disruptive lithographic technology has proven to be difficult. A new technology needs to address the fundamental resolution limits of PL while otherwise being a “drop-in replacement” to PL.

### Nanolithography literature review

The most aggressive half-pitch of the circuit patterns being manufactured today are about 20 nm half-pitch. Since the most advanced form of PL—193i lithography—is limited to ~38 nm half-pitch lines and spaces, the current production approach for sub-38 nm half-pitch patterning-self-aligned double (SADP)/quad patterning-comprises extremely complex and expensive processing steps^3^. Directed self-assembly (DSA) techniques are also being explored to complement 193i lithography^4,5^. SADP and DSA techniques are inherently suited for periodic patterns, and they place stringent constraints on device designers that require more complex patterns^6^. In addition to the patterning limits of DSA, it also has a number of challenges related to defects and pattern placement^7,8^, which has prevented its adoption to date in IC manufacturing.

Over the years, the industry has explored several other high-resolution lithography techniques for production including X-ray lithography (XRL)^9,10^, electron projection lithography (EPL)^11^, ion beam projection lithography (IPL)^12^, and 157 nm PL^13,14^. As of the writing of this document, XRL, EPL, IPL, and 157 nm PL are no longer being pursued by the silicon IC industry. In addition, multiple e-beam lithography (MEBL)^15,16^ is being considered for IC fabrication. MEBL has made progress towards the development of a master writing lithography tool for fabricating both photomasks and imprint templates. This definition of the term “template” has now been included earlier in the paper on page one in the section “semiconductor lithography requirements”. Wherein the write times for the most advanced photomasks are targeted to improve from several days to ~10 h (Refs. 17,18). However, a direct writing tool for wafer fabrication will require a further improvement in throughput by a factor of at least 30 000×. Current MEBL tools for mask/template writing are able to write a full-field (26 mm×33 mm) lithography region in ~5–6 h. To be able to write an entire silicon wafer consisting of about 100 fields in about a minute would require at least a 30 000× improvement in throughput.^19^ This will require hundreds of thousands of simultaneous e-beam columns that are individually addressable. This leads to highly complicated tooling and formidable technical challenges such as beam blur due to space-charge effects, and pattern-placement errors. Even if all these technical challenges can be addressed, MEBL’s throughput limitation will likely make the technology viable only for small volume ASICs, but not for high volume memory or logic applications.

Recent research in nanopatterning has continued to explore techniques that may become relevant in IC fabrication including plasmonic patterning approaches^20,21^, interferometric lithography techniques^22,23^, arrayed X-ray patterning with zone plates^24^, and arrayed tip-based patterning^25–29^. The references provided here are exemplar and not meant to be comprehensive. However, to the best of the author’s knowledge, none of the capabilities discussed in this paragraph have developed comprehensive systems that simultaneously address all the specifications listed in Table 1, nor have they reached the maturity to warrant a leading-edge IC manufacturing company exploring them.

As discussed next, while the research landscape for nanopatterning includes a variety of techniques, the only two technologies that are being explored and/or integrated into manufacturing by IC fabs to address resolution limits of 193i PL are J-FIL and extreme ultraviolet (EUV) lithography. These two technologies are the only ones that appear to have the maturity to be considered for production for sub-20 nm patterning with arbitrary pattern complexity, including, for example, lines/spaces and contact holes with arbitrary/variable pitch, and complicated circuit geometries.

#### EUV lithography

The historical trend in PL tooling has been to decrease exposure wavelength (λ) and/or increase the numerical aperture (NA) of the projection optics to exploit the diffraction limited resolution, *R*=λ/NA (Refs. 2,13). EUV lithography seeks to create enhanced resolution beyond 193i PL by using soft X-ray wavelength of 13.2 nm, and was originally explored by the industry in the early 1990s. While EUVL appears to be a natural extension of PL, it has several major challenges as compared to 193i PL, making it a disruptive extension of PL. EUV wavelengths cannot penetrate through any medium; therefore, EUVL tools operate in high vacuum. They can only incorporate reflective optical elements to create a projection lithography system and their photomasks are reflective elements as well. Creation of a viable EUV light source has been a monumental challenge as high-powered light sources (>200 W) are needed; every EUV reflective surface absorbs ~30% and there are typically at least seven reflective surfaces in the EUVL tool before wafer exposure^30,31^. EUV photons are usually created by subjecting a micro-droplet of tin to a high-powered CO_2_ laser. The state of the art today is a ~25 kW CO_2_ laser creating a 250 W EUV source. While EUVL was first proposed and explored in the early nineties, its use in manufacturing to achieve acceptable throughput for a reasonable cost of ownership has remained elusive due to the need for high-powered sources and other challenges such as contamination due to lack of practical EUV mask pellicles that can be used in a manufacturing setting^32^, complex reflective mask blanks that require multi-layer coatings consisting of ~80 atomically precise films, and pattern line-edge roughness (LER) due to the need for chemical amplified resists to achieve acceptable throughput for a reasonable cost of ownership^33,34^. Improvements in resolution and LER requires higher source power (due to the fundamental problem of shot noise) to retain adequate throughput. Delay in EUVL adoption creates a familiar problem in next-generation lithography: the need to overcome additional technology roadblocks for a future node where EUVL is projected to be adopted. For example, current projections for EUVL call for insertion at the 7 nm logic node, or more realistically at the 5 nm logic node^35,36^. Lapedus^37^ discusses the projected half-pitch associated with these nodes: “A hypothetical 7 nm finFET is projected to have anywhere from a 12 to 18 nm gate length and a 45 to 55 nm gate pitch, according to IBM. In comparison, Intel’s 14 nm finFET technology has a 20 nm gate length. It has a gate pitch of 70 nm.” Turkot^35^and Kim *et al*.^36^ indicate that source power requirements will likely double when the design rules are reduced to 5 nm. In addition, the next-generation ASML high NA tool (0.55NA) will only be a half-field tool to retain the 150 mm square mask form factor affecting productivity and hence cost of ownership. There are also new basic challenges such as three-dimensional (3D) diffraction effects caused by the 70 nm mask absorber layers, which do not exist in 193i PL. Also, the need for a EUV pellicle creates a major challenge in handling and thermal stability of an ultra-thin (sub-50 nm) polysilicon membrane whose operational temperature will likely exceed 600C^38^. Finally, systems engineering issues such as tool reliability will need to be addressed. For example, photons coming from increasingly higher power EUV sources can severely heat several sub-systems in the tool, leading to concerns about degradation of expensive components. Also, since the silicon wafer temperature has to be controlled to better than 5 mK for overlay purposes, heat extraction and management from vacuum pose significant engineering challenges.

#### Nanoimprint lithography

Nanoimprint lithography (NIL) techniques are known to possess remarkable replication capability down to sub-3 nm resolution^39^, and sub-7 nm half-pitch^40^. In this regard, NIL is unusual in the capability it offers as compared to other technologies discussed above; its resolution is unmatched, approaching molecular scale^39^. Additionally, as compared to PL—which has been the workhorse in the semiconductor and display fabrication industries—NIL’s resolution is largely unaffected by the field size being patterned, which has the potential to lead to high-throughput NIL processes. This combination of resolution and large area (high-throughput) of NIL has been demonstrated in the development of nanopatterning systems that can pattern: (i) full wafers including the substrate conformal imprint lithography system^41^ and the J-FIL-based Imprio 1100 System^42^; (ii) double-sided disks^43,44^, and rolls of flexible substrates^45–48^. These NIL systems have demonstrated the potential for sub-10 nm patterning at high throughputs. As an example, a double-sided nanopatterning system for hard disk drives has demonstrated both sub-10 nm patterning and >180 double-sided disks per hour^49^. A good overview of the various NIL tools that have been created commercially is provided in a recent book (see Table 2.1 of Ref. 50).

Translating the above molecular-scale replication resolution to a commercially viable IC manufacturing process, however, requires addressing a variety of process performance, cost, and reliability targets (Table 1). A number of mechanical patterning techniques including thermal molding NIL^51–54^, UV NIL^55–58^, and soft lithography^41,59^ have been discussed in the literature. As discussed next, these techniques are unable to address two critical requirements of IC fabrication—<10 nm overlay and the ability to address pattern complexity (Items 9–11 in Table 1). This establishes a need for an imprint technology that retains the molecular-scale resolution, while solving the overlay and pattern complexity problems.

The need for nanoscale alignment/overlay eliminates the use of thermal molding technologies that require elevated temperatures and pressure. Further, technologies that use polymer molds^41,59^ lack thermal and mechanical stability causing in-plane mold distortions that are likely unsuited for sub-5 nm overlay. Nanoscale overlay also requires the use of a step and repeat (S&R) patterning process, rather than a whole wafer printing process. This makes the use of “wet” UV curable spin-on films^56^ challenging as the wet films not only attract contamination as the wafer undergoes S&R process, they also cause premature curing of areas due to stray UV light. For leading-edge IC fabrication, the sub-5 nm overlay requirement and the need to match with 193i PL makes it extremely difficult to pattern full 300 mm silicon substrates in one step. Full wafer patterning causes higher-order distortions due to: (i) large area wafer topography; (ii) mismatch between various PL scanner fields; (iii) overall thermal stability of the wafer and template over the entire wafer that cannot be corrected without the use of very sophisticated arrays of real-time sensors and actuation systems; and (iv) fabrication challenges of 300 mm printing area templates. As discussed in section “J-FIL stepper system and associated imprint materials”, the current J-FIL stepper template format leverages the PL 6025 format as this substrate is available at reasonable cost, with very low defect levels, and are thermally stable to achieve sub-5 nm overlay. A one-step patterning of 300 mm wafers would require substrate infrastructure that does not exist today. The problem of large-area precision overlay has been addressed to some extent by exploring the use of arrays of thermal actuators^60^, but one-step full wafer patterning still requires significant innovation in sensors, actuators, and wafer-scale template fabrication technologies.

**Figure 1:**
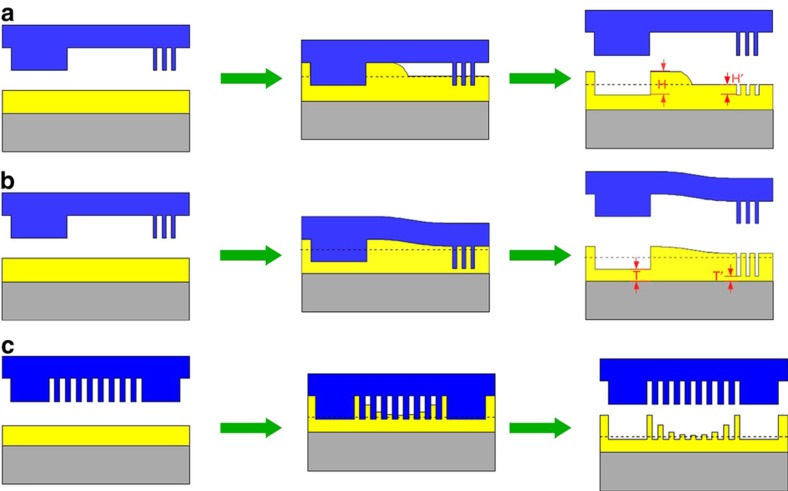
Various defects for the case of spin-on imprinting in the presence of pattern and size variation^61^; shear resistance can cause various defects, including non-filled features (**a**) and (**c**), and deformed features under pressure (**b**).

The ability to print complex patterns with pattern density variations eliminates the use of spin-coated resist films before nanoimprinting. This is because a non-uniform pattern in the template is incompatible with a uniform material distribution obtained on the wafer from spin coating as illustrated in Figure 1, which is reproduced here with permission from Cheng and Guo^61^.

J-FIL is a form of UV NIL that addresses the above two concerns. The next section describes the J-FIL process steps in detail, discusses its advantages, and the key technical risks. The third section presents the J-FIL stepper system architecture and related infrastructure choices made to address the key technical risks associated with J-FIL.

## Jet and flash imprint lithography

J-FIL is distinct from other imprint lithography processes as it uses inkjet techniques to dispense picoliter volumes of low viscosity UV curable resists. This enables adaptive material deposition to match pattern variations in the template. This, combined with the low viscosity resist formulations (<10 cP), leads to high-throughput processes^62–64^.

**Figure 2:**
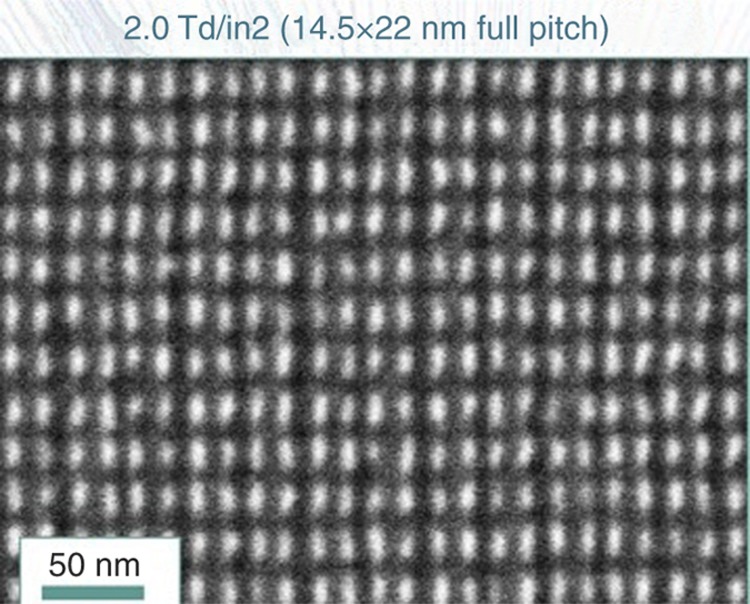
7 nm half-pitch patterns (data from HGST, a Western Digital Company^68^) using J-FIL. J-FIL, Jet and Flash Imprint Lithography.

J-FIL steppers pattern one rectangular field of ~26 mm×33 mm at a time (consistent with advanced PL steppers). Typically, about 100 fields cover a 300 mm wafer, including full fields and partial fields at the edge of the wafer. While this article focuses on J-FIL for semiconductor fabrication, J-FIL has also been deployed in the hard disk industry as double side full-disk patterning tools^65,66^, and roll-to-roll nanopatterning tools for display photonics applications^67^. In the hard disk drive area, J-FIL has demonstrated resolution below 10 nm over full disks as shown in Figure 2 (Ref. 68).

### Description of the J-FIL process

**Figure 3:**
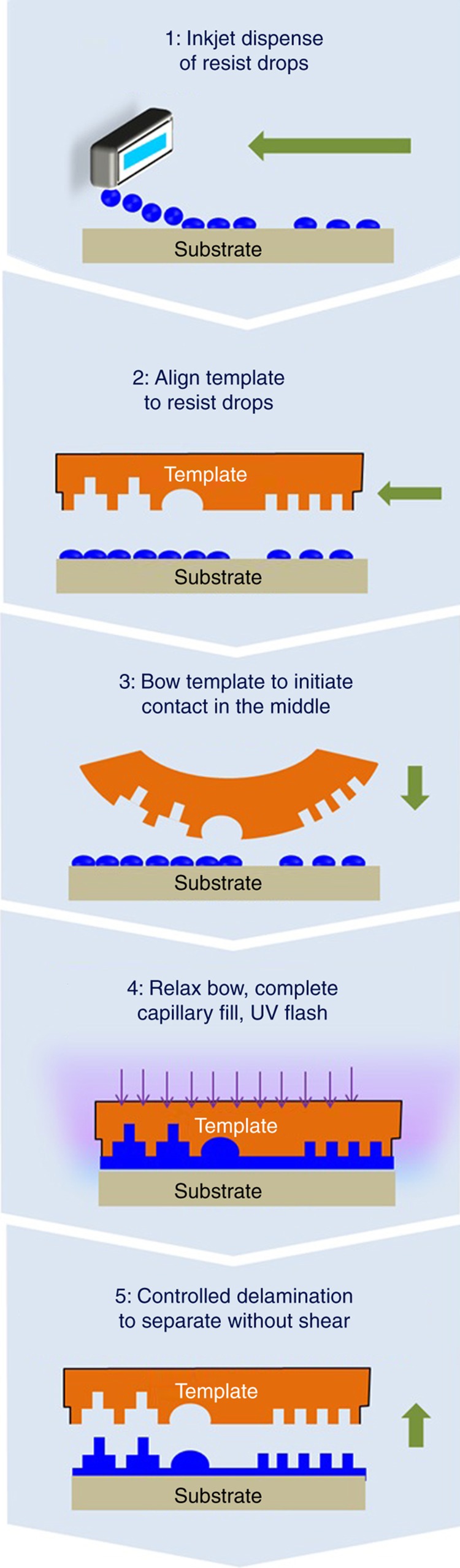
J-FIL uses inkjet-based adaptive material deposition to dispense resist material correlated with the template geometry, which can include binary, multitiered and generic 3D structures. 3D, three-dimensional; J-FIL, Jet and Flash Imprint Lithography; UV, ultraviolet.

The process steps are shown in Figure 3, and a summary of each step is provided below. It should be noted that these five steps have to be completed in ~1.5 s to achieve a commercially viable throughput of 20 wafers per hour (WPH) in a J-FIL stepper^69^.

**Figure 4:**
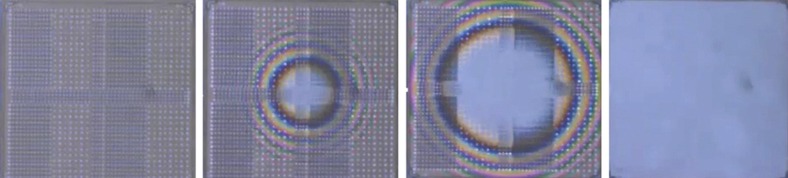
Steps 3 and 4 of Figure 3 shown here as “through-the-template snap shots” of the template engaging with the fluid. J-FIL, Jet and Flash Imprint Lithography.

The substrate is coated with a thin (sub-5 nm) adhesion layer (not shown in Figure 3) that promotes wetting of the liquid resist and provides strong adhesion to the cross-linked resist after UV curing^64^. Then, a low viscosity UV curable resist is dispensed onto the substrate. The dispensed geometry is based not only on the template pattern but also on the nature of fluid flow as a function of pattern type. For example, a grating-type pattern can cause highly directional flow, while a dot-type pattern leads to more isotropic flow^70^. The overall approach to drop dispense requires addressing many aspects including inkjet-to-inkjet variations, flow behavior at pattern transitions, the need to address evaporation of the liquid drops due to air flow in the equipment, and the fact that 10,000–100,000 drops having picoliter volumes may be dispensed over a field of 26 mm×33 mm. Offline software algorithms have been deployed to allow users to create the field drop patterns that address all these constraints^71^. The process steps shown in Figure 3 are:

1. The substrate is moved on the substrate stage to align the drops of resist accurately (better than ~5 μm) with respect to the template features to ensure efficient and precise fluid filling. The template is coated with a release layer to ensure that the adhesion at the template–resist interface is about 25 times lower than at the resist–substrate interface^64^, thereby avoiding resist contamination of the template.
2. The next step involves shaping the template with a bow of ~10–15  μm using air pressure to ensure that the template makes contact with the drops at its center (see Step 3 of Figure 3.)
3. Next, template bowing is relaxed using precise control of vertically moving actuators (see Figure 4). The speed of the template shape relaxation is also a function of the resist fluid properties such as viscosity and the fluid’s wetting behavior with the template and substrate. The speed is controlled to allow lateral merging of drops and capillary filling of features to minimize creation of trapped bubbles between the drops. Typically, if sub-micron bubbles are formed, they disappear quickly due to dissolution in the resist. If the desired fluid distribution is achieved, the resulting residual layer thickness (RLT) beneath the pattern is thin and uniform. To enable subsequent etching, the final residual layer has to have a mean thickness of ~1/2 to 1/4th the feature height. For a 20 nm feature that is 50 nm tall (aspect ratio of <2.5:1 is chosen to avoid defects during separation), the RLT mean ranges from 13 to 25 nm; and the uniformity needs to be ~3 nm, 3σ^64,72^. A reverse tone etch process can also be used with higher RLT mean; however, this process is not discussed here. Finally, before UV curing, nanoscale alignment/overlay relative to a prior pattern has to be ensured, followed by UV curing. This step, involving fluid filling and precision alignment, is the most time-consuming step and takes about 1.1 s^69^.
4. Step 5 consists of separating the template without shearing any nanoscale features, further aggravated by the need to complete the step in ~100 ms to achieve 20 WPH throughput.

### J-FIL stepper risks in semiconductor fabrication

The key J-FIL technology risks that were identified early in this project to enable advanced semiconductor manufacturing are listed below. Mitigation of these risks is addressed both conceptually and with recent results and data in the fourth and penultimate sections.

1. *Creation of 1X templates*: The introduction of 4X reduction PL in the 1970s was driven by the need to relax the fabrication and inspection requirements of photomasks. With the advent of deep subwavelength lithographic resolution, mask features are now significantly smaller than 4X, approaching 1.3X. J-FIL is a 1:1 replication of the template, that is,1X. This leads to challenges in the fabrication and inspection of the smallest and the tightest pitch structures. 4X masks also relax the tolerable image distortion requirements that affect wafer overlay by 4X, which is also a challenge for NIL templates. Solutions to this challenge are discussed in section “Resist and inkjet development strategy”.
2. *Template life*: J-FIL requires the templates to make liquid contact *via* the residual film between the template and substrate (typical mean value of <25 nm). Any hard particles (for example, inorganics or metallic) or other asperities larger than the residual film on the substrate can cause template damage leading to repeating defects. In PL, the pellicle is used to avoid such mask damage or contamination^32^. Solutions to this challenge in J-FIL are discussed in section “Resist and inkjet development strategy”.
3. *Basic S&R printing (precise fluid distribution and confinement within stepper full fields and partial fields)*: J-FIL’s low viscosity liquids can undergo significant undesirable/uneven evaporation if left uncovered on a substrate. Therefore, J-FIL steppers need to dispense one field at a time immediately followed by covering this field by the template to attain the process of Figure 3. This requires creation of perfect rectangles for each field (with no fluid extrusions outside the rectangular regions) followed by zero gap between a field and the subsequent field. This requires perfect fluid confinement within each field. Further, about 1/3rd of the stepper fields are “partial fields” at the edge of a 300 mm wafer. These partial fields contain >10% yielding devices and therefore it is essential that they are fabricated to meet all lithographic requirements. In J-FIL steppers, it is necessary to ensure that the liquid is confined in this asymmetrically shaped field to allow zero-gap patterning with adjacent fields, while avoiding resist extrusion over the edge of the wafer that can cause contamination and defects. This topic is discussed in section “Basic step and repeat printing (precise fluid distribution/confinement for stepper full and partial fields)”.
4. *Nanoscale overlay*: To be viable at ~20 nm half-pitch lithography, J-FIL needs to be capable not only of achieving sub-5 nm overlay while aligning to another J-FIL layer, but it also needs to “mix and match” with 193i PL and achieve sub-5 nm overlay. This requires rigid body alignment capability (*x*, *y*, and θ) to adjust scale or magnification (independent magnification in *x* and *y*), shape (orthogonality and trapezoidal), and often some higher-order distortion corrections as well. Nanoscale overlay is discussed in section “Nanoscale overlay”.
5. *Defect control:* In addition to particle-induced template repeaters, defects can occur in the liquid phase (before UV curing) or solid phase (after UV curing). Liquid phase defects include bubbles and voids, while solid phase defects include separation induced shear, cohesive failure of imprint materials, and feature collapse defects during or after separation. All these defects are discussed in section “Defect control”.
6. *Throughput*: Template fluid filling is known to be the bottleneck in J-FIL. Complete fluid filling is achieved only when all the bubbles/voids in the template–fluid–substrate sandwich have disappeared. Overlay control also happens in parallel to template filling. Steps 3 and 4 in Figure 3 and further detailed in Figure 4 represent the throughput bottleneck. These steps take up ~65–70% of the throughput budget^69^. Steps 1 and 2 take up <20% of the throughput budget and more importantly depend on mature technologies such as high-speed *x*–*y* stages with micro-scale precision alignment relative to the template. Step 5, while very important for defects as discussed below, only takes ~5% of the throughput budget. Steps 3 and 4 are discussed in section “Throughput”.

## J-FIL technology translation strategy

The development and deployment of J-FIL stepper technology for semiconductor fabrication required several strategic decisions during the early phases of the project. First and foremost was the decision to develop a stepper with the maximum field size matched with the PL standard field of 26 mm×33 mm. The next decision was to choose an appropriate initial target market and an initial customer. Then, a partnership was needed to create the template infrastructure, followed by a strategy to develop the J-FIL resist materials. Finally, a partnership to develop a high volume manufacturing (HVM) stepper was pursued.

It is worth noting that the market for silicon ICs is large, estimated at over U$330B in 2016 (Ref. 73). This readily supports a vibrant ecosystem of suppliers that contribute to the process technology in semiconductor fabrication facilities. Companies in this ecosystem create and deploy extremely sophisticated products in areas such as fabrication equipment for patterning, vacuum processes, wet processes, and so on; processing gases and materials; photomasks for lithography; ultra-low defect 300 mm crystalline silicon wafers; and so on. These suppliers play a critical role in enabling nanometer scale precision over macroscales; high yields; and high throughput exemplified by the lithography requirements in Table 1. In this section, the way this ecosystem was leveraged during the development of J-FIL technology is discussed.

### Stepper decision

J-FIL as well as other imprint processes can be, and have been, used to pattern the whole wafer at once which significantly enhances productivity^63,74,44^. This is a major advantage of NIL as compared to PL that is limited in its exposure field size by the need for extremely sophisticated optics. However, when it comes to semiconductor IC fabrication, the following aspects make S&R patterning desirable even for J-FIL:

- *Leveraging photomask infrastructure*: The semiconductor industry has developed the photomask blank to meet stringent material stability and defect requirements at acceptable blank costs. These blanks inherently support about 100 mm×100 mm area for mask patterns. However, since templates require additional features (such as a cored out region, see section “The stepper system”), the J-FIL field size has to be limited to about 50 mm×50 mm. The current industry ecosystem does not support the creation of templates large enough to support one-shot full wafer (300 mm) patterning.
- *Precision overlay*: Over full wafers, it is very difficult to avoid parasitic overlay errors and distortions as many errors degrade when the printed field is increased. For example, lack of substrate flatness, lack of temperature control, and overlay errors due to magnification and rotation errors are all aggravated when the field size is large. Therefore, an S&R process is preferred.
- *J-FIL integration with PL*: To leverage previously installed PL tools in the fab, integration of J-FIL (used for a few advanced layers) with PL is needed. By using a field size that is equal to a PL field (26 mm×33 mm), this integration is enabled while achieving sub-5 nm overlay.

### Initial target market

J-FIL is inherently better suited for advanced memory (non-volatile memory including flash and cross-point memory, and DRAM) manufacturing as memory manufacturers have tight cost constraints, and have relaxed defect requirements as compared to logic circuits such as micro-processors. The acceptable defect density for memory is ~1–10 defects cm^−2^, which is ~2–3 orders of magnitude higher than what is needed in logic circuits. Within memory, the first target market for J-FIL was chosen to be flash memory, which has somewhat relaxed overlay requirements as compared to DRAM. Toshiba—a leading flash memory manufacturer—became the first customer to investigate J-FIL technology for IC fabrication^75^; and SK Hynix—a leading DRAM manufacturer—joined Toshiba in developing J-FIL for manufacturing^76^.

### Template infrastructure partner

J-FIL templates require 1X patterns and are prone to template life problems due to potential for particle-induced template repeaters as discussed in the second section. While photomasks have historically been 4X, the advent of optical proximity correction sub-resolution features and computational lithography^77^ have led to the need for minimum mask feature size of <1.3X over 16X the area of imprint templates. Therefore, the original advantage of 4X mask patterns introduced in the 1970s to make mask fabrication easier is no longer true. Therefore, the electron beam write times for templates is comparable to or often lower than that of advanced photomasks. A careful comparison of write times for imprint templates versus photomasks is discussed in the literature^78^, and this topic is not discussed in any further detail in this article. Dai Nippon Printing Co. (DNP) in Japan, a leading-edge photomask manufacturer, has been the exclusive partner for J-FIL stepper templates over the past 10 years and has delivered commercial grade templates by leveraging its existing photomask fabrication facilities. In addition to achieving reasonable write times for imprint templates, DNP has used thinner chrome layers (<10 nm thick) since the chrome film is only being used as an etch mask in templates rather than for optical opacity in photomasks. This, along with non-chemically amplified electron beam resists, have enabled resolution of J-FIL templates to be well below 20 nm half-pitch for both lines/spaces and contacts with <1.4 nm, 3σ pattern uniformity^19^. DNP has also demonstrated very low image distortions (<1.5 nm, 3σ), thereby delivering the templates required for sub-5 nm overlay.

The issue of template life is related to template damage due to hard particles. This has been addressed by two approaches, first template replication discussed in this paragraph and second particle reduction in HVM stepper tools (see section “Particle contamination and template life”). Template replication exploits the fact that a J-FIL template is 1:1 and therefore a master template created by e-beam can be duplicated to create replicas inexpensively using J-FIL-based template replication tools. Template replication is a critical technology that supports the J-FIL stepper infrastructure. As will be discussed in section “Particle contamination and template life”, the current template life can extend to >1000 wafers because of the extremely low particle contamination counts that are observed today in J-FIL steppers. Hence, a replication strategy involving one e-beam master being used to make >100 replicas, followed by each replica patterning >1000 wafers leads to an equivalent master template life of >100 000 wafers, which meets manufacturing cost-of-ownership requirements. Template replicator tools were first created by Molecular Imprints Inc. (MII)^79^, and then further developed by Canon Corporation, resulting in the replica production tool, FPA-1100-NR2^80^. DNP has used J-FIL-based template replicators and demonstrated their ability to provide commercial template replicas that meet resolution, pattern uniformity, image placement, and defect requirements. Template replication tools and processes are not discussed any further in this article.

### Resist and inkjet development strategy

In PL, photoresist materials are spun on to wafers and then processed through 193i PL tools. The industry has evolved to a decoupled development approach, wherein the PL tool manufacturers (ASML, Nikon and Canon) develop the tools while materials companies (JSR, EMD Performance Materials, TOK, Fuji Film, and so on) develop the photoresists. During the development of J-FIL, this decoupling between tool and material supplier was not pursued as the inkjetting approach incorporates the material dispense capability in the stepper (eliminating the need for a resist spin coater), and the unique control algorithms needed to operate the inkjets at sub-picoliter volumes requires considerable co-development between the tool and the resist materials. Additionally, the inkjet approach leads to a near-zero-waste process as compared to spin coating, which uses solvents that form a majority of the as-formulated resist volume, and the spin coating process creates a significant waste stream as the materials spins off over the edge of the wafer. J-FIL therefore requires ~1% of photoresist volumes. Since resists are typically priced by volume, very low J-FIL resist volumes create a business dis-incentive for existing material suppliers. For these reasons, resist development has been integral to the stepper development in MII and subsequently in Canon Corporation. Additionally, a long-term partnership with Xaar^81^, which has leveraged their high-speed industrial grade piezo inkjet arrays to create a semiconductor grade solution for J-FIL resists, has been critical to the success of J-FIL steppers. This partnership has resulted in: (i) reliable jetting of sub-picoliter resist volumes at sub-5  μm placement accuracies, and (ii) jetting of imprint resist materials with parts per trillion contamination of metal ions that are undesirable in semiconductor fabrication.

### HVM stepper development partnership

The J-FIL stepper development included prototypes developed at UT-Austin and intermediate products developed at MII achieving several technology milestones before the development of an HVM tool. This technology and product evolution is discussed in the last section. The HVM tool development was a significant effort as the tool had to not only achieve the metrics discussed in Table 1, it also had to be suitable for a leading-edge memory fab. The tool therefore had to meet stringent engineering reliability requirements, footprint constraints, and seamless integration into a process flow in the fab. As leading-edge semiconductor fabs cost billions of dollars to build, a tool failure can cause a process line to go down resulting in significant revenue losses. A partnership between MII and Canon was created to leverage the J-FIL technology know-how in MII and PL stepper experience in Canon. A novel strategy was chosen to jointly develop the HVM stepper involving:

- The creation of an imprint module (IM) that captures the J-FIL core technologies such as precision resist jetting, real-time alignment nanometrology, magnification and shape control system (MSCS), template shape control system, and separation system.
- Integration of the IM into a “stepper platform” to create a J-FIL stepper. The stepper platform includes a nanoprecision, high bandwidth *x*–*y *stage; sub-10 mK temperature control; particle control; and automation of wafer and template handling.

**Figure 5:**
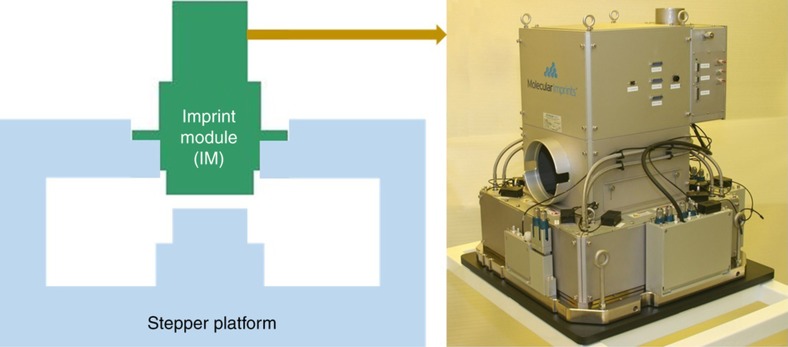
Illustration depicting the integration of the IM from Molecular Imprints Inc. in Canon’s stepper platform. IM, imprint module.

This partnership approach (illustrated in Figure 5) allowed the J-FIL process to be deployed in the fab, and eventually led to the acquisition of the semiconductor unit of MII by Canon in 2014.

## J-FIL stepper system and associated imprint materials

To meet desired lithographic specifications, the J-FIL technology risks listed in section “J-FIL stepper risks in semiconductor fabrication” (risks 1–7) need to be addressed. Risk 1 and part of risk 2 have been addressed in section “Initial target market”. In this section, the key J-FIL building blocks required to overcome the rest of the risks 2–7 are addressed. The key building blocks are: (i) J-FIL stepper system; and (ii) the materials (resist and adhesion layers) associated with the J-FIL process. These two building blocks are discussed next. The next section then discusses how these building blocks address technology risks presented in section “J-FIL stepper risks in semiconductor fabrication”.

### The stepper system

**Figure 6:**
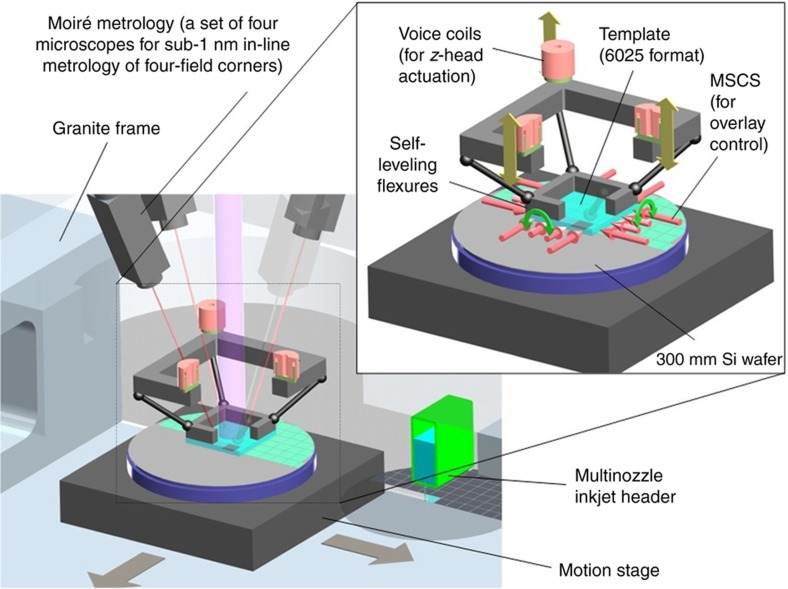
Illustration of some critical modules of the J-FIL stepper system. The modules shown include multi-nozzle piezo inkjets, nm-accuracy motion stage, template MSCS, i-MAT, and tilting or self-levelling flexures for the template. i-MAT, interferometric Moiré alignment technology; J-FIL, Jet and Flash Imprint Lithography; MSCS, magnification/shape control system.

As shown in Figure 6, a J-FIL stepper system includes precision mechanisms, such as self-leveling flexures for controlling the motion of the imprint template chuck^82^; nanometer-precision vacuum pre-loaded air-bearing motion stages for moving the wafer; interferometric Moiré-based alignment technology capable of <1 nm alignment measurement resolution^83–85^; piezo-based MSCS for scale and shape-change deformation of the template for meeting overlay requirements^86,87^; and piezo “drop-on-demand” inkjets with nozzle arrays having sub-picoliter volume control of UV curable resists^88^.

**Figure 7:**
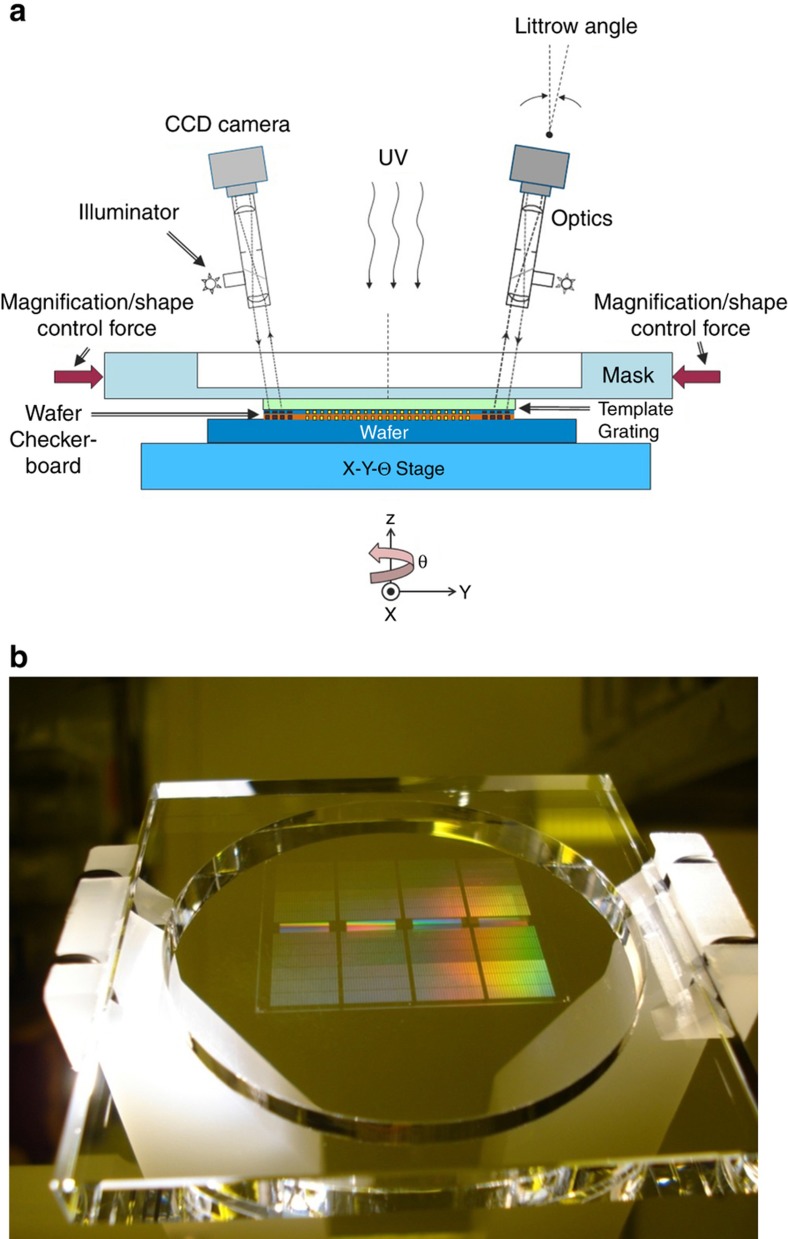
(**a**) An illustration showing the template, wafer and the i-MAT system on 6025 glass showing the circular cored out. This figure is reproduced with permission from Cherala *et al*. (Ref. 87). (**b**) A photograph of an imprint template on 6025 glass showing the circular cored out region and the 26 mm×33 mm pattern area. i-MAT, interferometric Moiré alignment technology.

Figure 6 shows the *x*–*y*–θ stage that has a motion range of >350 mm in *x* and *y* and few milliradians in θ; and a resolution of ~1 nm in *x* and *y* and ~50 nrad in θ. This stage scans the wafer underneath the resist jetting system, then aligns the drops to the template as well as the template and wafer alignment marks to within about 500 nm (Figure 3, Step 2) using the interferometric Moiré alignment technology (i-MAT) system (see i-MAT system details shown in Figures 6 and 7).

Next, the template is deformed to create a convex shape to engage the discrete drops near the center of the template field (Figure 3, Step 3). The J-FIL template leverages the industry standard “6025” format: 6025 stands for a standard fused silica photomask substrate that is 6″x6″ square and 0.25 in thick (152 mm×152 mm×6.35 mm). These substrates meet the stringent defect and material property requirements (optical and thermomechanical stability) needed for PL as well as J-FIL for IC fabrication. The 6025 glass blank is customized for J-FIL by machining it to create a cored out region directly behind the patterned area so as to create a thin section that is about 1 mm thick (see template in Figures 7a and b).

This thin section of the template allows for micro-meter-scale parabolic deformation of the template via pressurization and/or mechanical means (not shown in Figures 3 and 6 or Figure 7) before touching the resist drops. This deformation causes a convex surface to contact the drops and enable the formation of a contiguous liquid film from the discrete drops without forming bubbles (see Figure 3, Step 3)^63,64^. The three voice coils provide precise up-down (*z*-direction) motion (for imprint and separation) enabling the template to relax its corners and complete the fluid filling step (see Figure 3, Step 4 and Figure 4).

**Figure 8:**
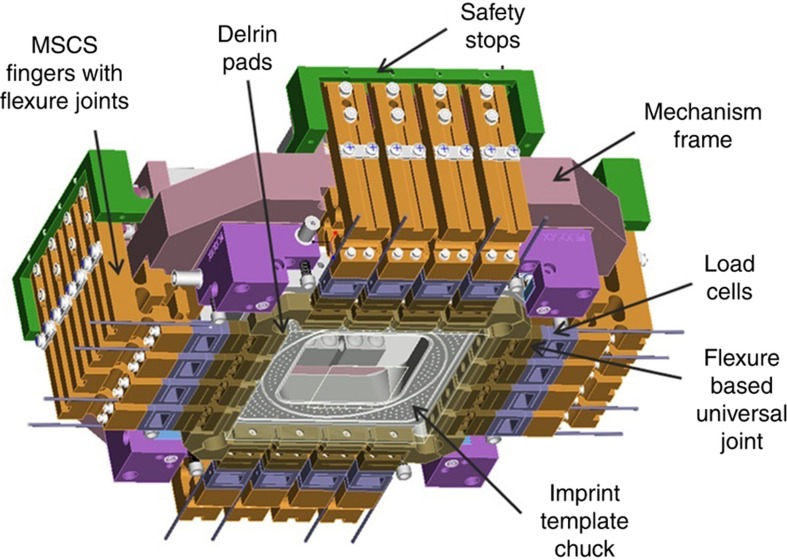
Isometric view of the magnification/shape control system (MSCS). This figure is reproduced with permission from Cherala *et al*.^87^

The template’s first engagement with the drops is detected using force sensors and then the precision alignment process is initiated. This includes using the i-MAT system (shown in Figures 6 and 7) to detect the *x*–*y* alignment errors at the four corners (eight scalar errors) to <1 nm resolution. After the template has completely relaxed from its bowed shape, the eight errors sensed by the i-MAT system can be used to compute errors in *x*, *y*, θ, magnification-*x*, magnification-*y*, trapezoid-*x*, trapezoidal-*y* and orthogonality^87^. These eight errors are corrected by the *x*–*y*–θ stage combined with the MSCS that corrects the other five errors. The MSCS is shown schematically in Figure 8. It comprises 16 actuators, four for each side of the template, each actuator combined with a six-bar linkage flexure that provides the desired force and displacement output to the template. (Flexures are compliant joints with sub-nm resolution that work by inducing strain energy in the joint. They are used in semiconductor industry as they possess no friction or backlash and do not generate particle contamination.) The end of the six-bar mechanism is fitted with a load cell on each finger to sense the end-point force. A pad made of compliant Delrin (a DuPont material) is connected to the load cell through a universal flexure joint. The Delrin pad contacts the glass template with the center of the contact surface nominally in line with the center of the template side, to apply a force normal to the side wall. The compliant material avoids stress concentration points at the contact surface. The universal joint provides tip-tilt degrees of freedom for the pad to conform to the template surface. This ensures uniform surface contact between the pad and the template. It is important to note that during the magnification control step, out-of-plane bending of the template can occur due to the cored-out region shown in Figure 7. This out-of-plane bending is however completely eliminated during the “in-liquid” phase of the imprinting process, because the thin liquid layer (<25 nm mean thickness) holds the template flat against the wafer surface due to the very high capillary forces (see Step 4 of Figure 3). Therefore, it is critical that the MSCS be actuated to correct for the magnification/shape errors only in the in-liquid alignment phase (Step 4 of Figure 3).

A schematic of the alignment sub-system, based on an i-MAT, is shown in Figure 7a. The alignment marks constitute a phase grating pattern on the template and a phase grating checkerboard on the wafer^83,86,87^. Upon illumination and inspection under a microscope, the grating patterns form a moiré pattern along the main axis, *X*, that is perpendicular to the plane of Figure 7a. By illumination along the Littrow angle, which eliminates noisy zero-order interference, the signal becomes insensitive to the gap variation between template and wafer, thereby providing a completely decoupled in-plane alignment error. The use of sophisticated image/signal processing then detects misalignment errors with a resolution of <1 nm and at >500 Hz. Using these i-MAT alignment errors, the *x*, *y*, θ, magnification-*x*, magnification-*y*, trapezoid-*x*, trapezoidal-*y*, and orthogonality errors are corrected by the *x*–*y*–θ stage combined with the MSCS to achieve precision overlay immediately before UV exposure in Step 5 of Figure 3.

In the state-of-the-art steppers from Canon, the i-MAT system has been further improved to create a through-the-template microscope system^69^; and the MSCS system has been recently complemented by a higher-order distortion correction (HODC) system^69^ enabled by precision, selective heating of the wafer field being imprinted using a laser source and a DMD system. The DMD (digital micro-mirror device) is a micro-opto-electromechanical system projection technology from Texas Instruments^89^.

Piezo ‘drop-on-demand’ inkjets enable adaptive material distribution (Step 1 of Figure 3) that allows imprinting of patterns of varying densities with the same RLT. Piezo inkjet arrays that can dispense sub-1 pL drop volumes of the resist material are used for this purpose. These inkjet arrays have ~360 nozzles per inch in a single inkjet head, or ~720 nozzles per inch obtained by interleaving two inkjet heads. These jets cover the entire width of a field. They can scan across a field (by scanning the *x*–*y* stage that carries the wafer) and dispense <1 pL drops to within 3 μm positioning accuracy relative to an ideal grid at speeds exceeding 1 m s^−1^. Further, these inkjet heads have been demonstrated to perform reliably (without defects such as missing or deviated nozzles, loss of volume control, or loss of placement accuracy) in excess of 6 months on J-FIL steppers.

### Imprint materials

Imprint materials, including the imprint resists and the substrate adhesion materials, are important building blocks of the J-FIL stepper process. The primary function of the imprint resist is to form a polymer replica of the template. This replica forms a sacrificial patterned material that serves as an etch mask during subsequent reactive ion etching that transfers the pattern to an underlying film or film stack on the wafer. The adhesion material serves to modify the surface properties of a wafer to promote wetting during the liquid phase prior to UV curing. It also promotes adhesion of the imprint resist to the wafer during the solid phase after UV curing. The development of these materials is discussed next.

#### Imprint resist

The imprint resist is a material composition comprising of several constituents designed to achieve desired process considerations. Some of the process considerations can place conflicting requirements on the composition requiring a good understand of the trade-offs. The imprint resist is dispensed as discrete drops using inkjets in a liquid formulation, the liquid is captured in the template features, and finally the liquid is cross-linked using UV light to polymerize into a solid material. Hence, the resist must have desired liquid and solid phase properties. The liquid phase properties include density, viscosity, surface tension, contact angle, and volatility. On the other hand, the solid properties include cohesive yield strength, shear strength, elongation to break, adhesion to substrate, and ease of release from template. The optimum material formulation involves a consideration of all these characteristics.

The liquid properties of the resist are primarily tailored towards robust inkjettability of picoliter volumes, low parasitic evaporation, and fast defect-free wetting of both the substrate surface and the template features. Low viscosities (~1–5 cP) generally assist dispensing and fluid filling, but can have high volatility, which can cause inconsistent cured resist behavior. Hence, fluid viscosity and evaporation properties need to be carefully balanced. When all template patterns are fully filled, the UV cross-linking step follows. During UV exposure, the material needs to cross-link quickly for high throughput and to prevent thermal loads on the substrate and template. Thermal loads can compromise overlay by causing parasitic expansion and nanoscale in-plane distortion of the patterns. Next, during the separation step, it is necessary to ensure that the material adheres to the substrate and not to the template, which is a conflicting requirement when compared against the liquid phase property of requiring wetting and filling of the template features. Separation also requires a material with adequate mechanical strength, toughness, and Young’s Modulus to maximize the aspect ratio that can be patterned and yet completely prevent the possibility of a feature being distorted or left in the template. Adding polar resist components helps with these properties, but excessive amounts increases the surface tension and reduces the fill speed in the fluid phase.

Many types of UV curing chemistries including methacrylate, epoxy, vinyl ether, thiol-ene, and acrylate have been considered for imprint resist. Methacrylate and epoxy have considerably slower polymerization rate. Hence, they are not practical for high-throughput volume manufacturing. Most of the UV imprint materials are acrylate-based materials. Vinyl ether, which has low viscosity, relies on cationic polymerization to cure the liquid resist. It is inherently not inhibited by oxygen, which scavenges radicals. On the other hand, the cationic initiator is sensitive to the presence of excessive moisture and has concerns related to shelf-life stability as well as interaction with fused silica templates. Thiol-ene provides a promising alternative to acrylate chemistry for UV imprint resists. It has distinct advantages over acrylate systems including low oxygen inhibition during cure and low separation force against fused silica template. The disadvantages of thiol-ene systems are strong odor of thiol, and limited amount of commercially available compounds. The shelf-life of thiol-ene systems can be extended by adding a stabilizer. In summary the leading choice of polymerization chemistry for imprint resists is acrylate materials. Acrylate materials possess good curing speed, and are typically one order of magnitude faster than methacrylate chemistries. It is also advantageous that many different acrylates are commercially available, which readily enables testing of many formulations of imprint resists with varying parameters such as viscosity, surface tension, and solid mechanical properties. Acrylates also have excellent shelf-life lasting at least 6 months, which can possibly be extended to 12 months. The primary limitation of acrylates is that it is susceptible to oxygen inhibition, which needs to be balanced.

**Figure 9:**
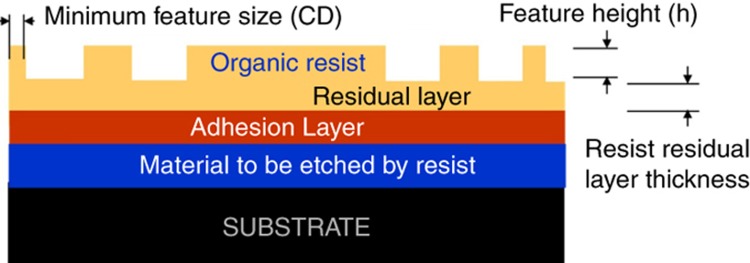
J-FIL material stack showing silicon-free organic resist and adhesion layer. J-FIL, Jet and Flash Imprint Lithography.

Early resist formulations included silicon to enable high etch resistance in oxygen^90^. However, silicon containing materials, if trapped in the template during a process excursion, can cause template cleaning issues as they form silicon dioxide easily in small features and are then hard to remove chemically. Later resist formulations therefore used silicon-free organic materials^64,91^, and used the film stack shown in Figure 9. Overall, J-FIL resists used today possess a viscosity of about 7–10 cP, contact angle with the template of <30°, contact angle with the wafer (coated with adhesion layer) of <5°, UV dose to cure of about 75 mJ cm^−^^2^ (UV cure time of as low as ~100 ms), cured modulus of >1.3 GPa, material strength of >25 MPa, resist elongation to break of >20%, and etch behavior similar to 193i PL resists. Typical maximum aspect ratio is (*h*/CD)≅2.5 (see Figure 9).

#### Adhesion material

The fidelity of the J-FIL stepper process is influenced by many solid phase resist characteristics including preferential adhesion ratio defined as the ratio of adhesion between the resist material and the adhesion layer (*A*_1_) on the substrate divided by the adhesion between the resist material and the template surface treated with a release layer (*A*_2_). To increase this preferential adhesion ratio (*A*_1_/*A*_2_) for a given release layer on the template, an optimal adhesion layer or prime layer is applied to the substrate. The adhesion layer should be quite thin (for example, 1–2 nm) as it adds to the residual layer. If the residual layer is ~15 nm, and the adhesion layer is ~2 nm, the etch process needs to first “break-through” the 17 nm organic underlayer in a non-selective, anisotropic reactive ion etch (RIE) step. This RIE step has a strong physical component that can damage the top of the resist feature. To maintain a good resist mask after the break-through etch, the starting resist height (*h*) needs to be 2–3 times the thickness of the underlayer (residual and adhesion layer combined). The adhesion layer is typically deposited onto the substrate via spin coating or vapor treatment methods. This 1–2 nm thin film must not include any pin holes or contaminants that could cause defects during imprinting. The adhesion layer also needs to be engineered in such a way that it can work on typical substrates such as Si, SiO_2_, SiN, and so on, and be readily wettable by the imprint resist in its liquid form. A spin-coatable adhesion material (known as TranSpin)^64^ has been custom designed for the J-FIL process. It undergoes covalent bonding with the resist during UV curing, and provides *A*_1_/*A*_2_≅30. TranSpin has demonstrated robust J-FIL processes at <10 nm half-pitch resist patterns^66,68^.

## Overcoming technical risks

This section describes the role of the J-FIL stepper system and the imprint materials in overcoming the technical risks identified in section “J-FIL stepper risks in semiconductor fabrication”. Risks associated with template fabrication (risk 1 and a portion of risk 2 associated with template replication) were discussed in section “Initial target market”; these risks are not discussed here. The rest of the risks are addressed below.

### Particle contamination and template life

**Figure 10:**
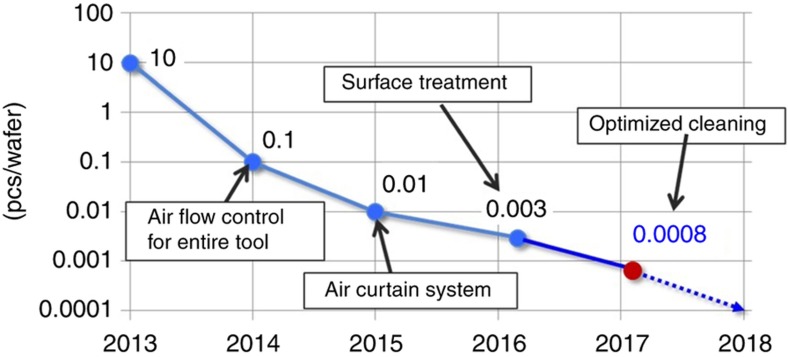
Reduction in tool particle density over 5 years.

As discussed in section “J-FIL stepper risks in semiconductor fabrication”, hard particles (for example, inorganic materials such as SiO_2_, SiN, SiC, and metals) that are larger than the RLT can cause template damage. PL benefits from pellicles that protect the mask from being contaminated and causing repeating defects; however, EUV lithography currently does not have such a pellicle as discussed in section “EUV lithography”. The strategy chosen for J-FIL is one where a template replica can pick up a small number of repeaters as long as the total defectivity stays below what is expected in memory applications. Typical cost models for J-FIL (see for example Ref. 69) require processing of >1000 wafers per replica, wherein the replica repeater defect density does not exceed ~5 defects cm^−^^2^. Here, a total defect density of 10 defects cm^−^^2^ is assumed to be acceptable for contact layer in memory applications as discussed in section “Stepper decision”. Recent results^92^ indicate that template repeaters are almost exclusively caused by particle events that damage the template; and ~0.0008 particles per wafer (indicating 1 particle event every ~1250 wafers) has been achieved (see Figure 10 that has been reproduced with permission from Ref. 92). This is a significant achievement as the particle levels are 2–3 orders of magnitude better than best practices, even in the semiconductor fabrication domain. This particle reduction effort was performed using a KLA-Tencor Surfscan SP3 tool. This tool is capable of identifying particles that affect 2x nm memory device patterning^93^. (Here 2x nm refers to 20–29 nm half-pitch device design rules.) A multifaceted particle control approach, both in the tool and in the resist materials, has resulted in this capability. Particle reduction in the resist, down to 0.02 particles ml^−1^, was achieved using a dynamic recirculating filtration system consisting of two separate filters with <5 nm sized pores. This recirculating filtration approach is needed as, at sub-20 nm levels, many of the components of the resist delivery system are known to shed low levels of particles (on the order of parts per billion) as pressurized resist monomer liquids move through them. In the tool, particles can result from air flow, from ceramic material surfaces, and from moving components, particularly the ones involving frictional contact. System design and material choices that avoid particles include: (i) sophisticated air curtain systems; (ii) surface treatment of ceramic surfaces; and (iii) exclusive use of non-contact motion systems such as air bearings, magnetically levitated systems, non-contact electrical actuators (voice coils, linear motors, and so on) and flexure mechanisms discussed in section “The stepper system”. A detailed treatment of this topic is included in Ref. 92.

### Basic step and repeat printing (precise fluid distribution/confinement for stepper full and partial fields)

**Figure 11:**
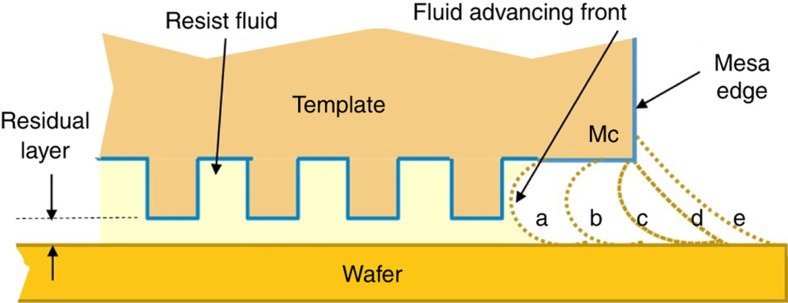
Illustration of template and wafer with the resist fluid between them, wherein the advancing front of the fluid approaches the mesa corner (*M*_c_).

Two important aspects of a viable J-FIL stepper process are described next. First, *the need for a thin and uniform residual layer* is discussed. As described in section “Description of the J-FIL process”, accurate fluid distribution across the field is needed to match the pattern density variations in the field to achieve thin and uniform residual layer (see Figure 11). To enable subsequent etching, the mean thickness of the final residual layer has to be <~1/2 to 1/4th the feature height. It is important to note that when the template engages with the resist monomer fluid drops, the local fluid flow is a function of the template patterns. Grating structures can cause highly anisotropic flow patterns, while dot patterns result in isotropic flow. Ideally an inverse optimization scheme that is based on comprehensive forward models, is needed to correctly identify the volume and placement of the monomer drops. Heuristic algorithms have found success in addressing this problem^71^, while more comprehensive model-based approaches have been pursued^94^ with some success.

Next, methodologies used to *avoid fluid extrusions beyond the full rectangular field and partial fields* are explained. Precision fluid confinement at the field boundaries is needed to enable patterning of precise field rectangles with no fluid extrusions outside these rectangles. There must also be zero gap between a field and the subsequent field (as discussed in Step 3 of section “J-FIL stepper risks in semiconductor fabrication”), thereby creating a continuous patterned polymer film akin to patterned resist films achieved by PL. This is achieved by taking advantage of “capillary pinning” at the edge of the field being imprinted. The template format includes a “mesa” (see Figure 7a and Figure 11) on which the nanoscale patterns reside. The mesa has the same size as the desired rectangular field (maximum size of 26 mm×33 mm), and includes a sharp edge (*M*_c_ in Figure 11), which restricts the motion of the fluid front from beyond the desired rectangular field. As discussed in section “Imprint resist”, the liquid resist forms a contact angle of <30° degrees with the template, and a contact angle of <5° with the wafer (coated with adhesion layer). The wetting front on the template approaches and arrives at *M*_c_ (liquid fronts “a” through “c” in Figure 11). Then, the liquid has to circumvent the right angle at *M*_c_ requiring it to go from front “c” to “d” and eventually “e”. If front “e” is reached, the liquid is assumed to have extruded beyond the field which is undesirable. However, the transition from “c” to “d” can take a few seconds, providing a “pinned” fluid front which provides a time window when UV curing can capture a perfect rectangular region. At the edge of the wafer, an exclusion area of 2 mm or higher is used by industry. This region provides a buffer zone that tolerates significantly less precise definition of the liquid front—for example, tens of microns of variation—as compared to the zero-gap requirement between fields. In addition, pinning similar to the one shown in Figure 11 can also be achieved at the wafer edge by having a sharp transition in cross-section profile on the wafer side. As discussed in the literature^95,96^, there are two major types of silicon wafer edge cross-section profiles: (i) blunt-nosed (rounded) edge; and (ii) bullet-shaped (beveled) edge. The latter includes a sharp corner at the onset of the beveled surface and can cause liquid pinning. Additionally, the wafer edge needs to be prepared to avoid any edge beads resulting from thin film deposition processes that result in raised regions at the edge of the wafer as this will invariably cause the template to interfere with this edge bead causing disruption of the edge field patterning process.

**Figure 12:**
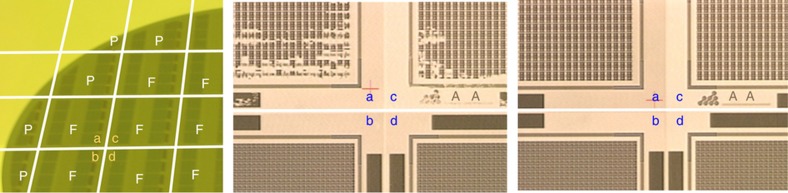
The left image shows a picture of a J-FIL patterned wafer with field boundaries superimposed on it to identify the full field (F) and partial field (P); the middle image is an optical micrograph taken at the interface of four full fields indicated by “a–b–c–d” in all three images, wherein fields “a” and “c” possess void defects due to inaccurate fluid dispense; and the right image shows the same four fields after a process calibration step that eliminates the void defects. J-FIL, Jet and Flash Imprint Lithography.

**Figure 13:**
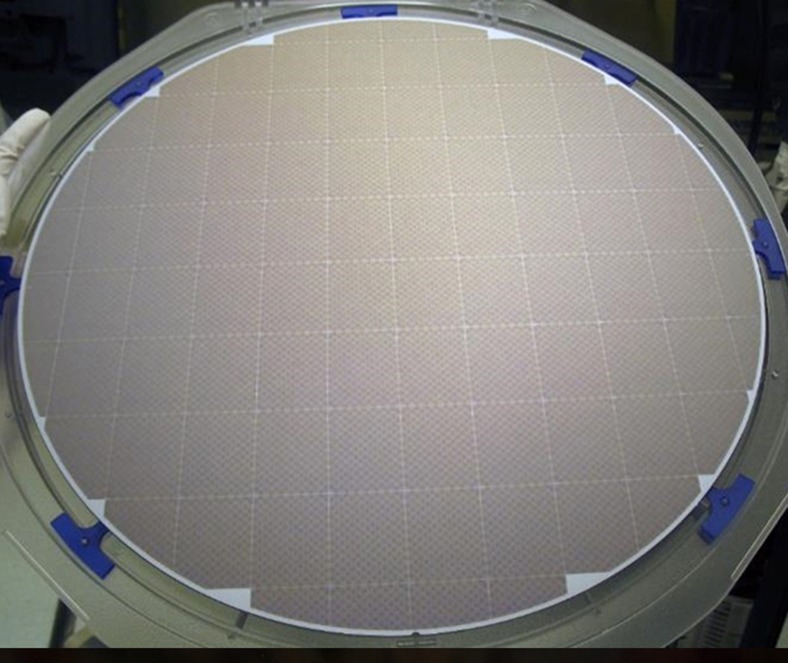
Fully patterned 300 mm wafer using a J-FIL stepper. J-FIL, Jet and Flash Imprint Lithography.

Figure 12 shows a photograph of a portion of a J-FIL patterned wafer (left), and two optical micrographs of four full fields (fields “a–b–c–d”) with zero gaps between them. As shown in the right image of Figure 12, four fields can be printed with zero gap between them and without void defects (voids are seen in the middle image of Figure 12). In the right image, slight color variations still exist between the four fields (for example, fields “a” and “c”) indicating small height variations. The height variations have been shown to be <3 nm. Such small variations do not appear to affect subsequent etch processes. Figure 13 shows a photograph of a fully patterned 300 mm wafer using the J-FIL process demonstrating liquid confinement and zero gap between both full fields and partial fields.

### Nanoscale overlay

**Figure 14:**
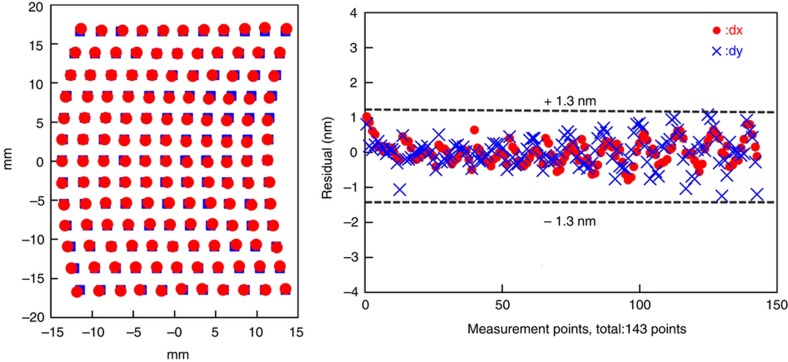
Higher-order distortion correction demonstrated for K11 bow-shaped distortion over a 26 mm×33 mm J-FIL stepper field based on 143 overlay errors (13×11 array) measured by KLA-Tencor Archer-500 Metrology tool; left: HODC system results showing target locations (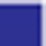) and experimentally achieved locations (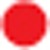); right: residual distortions between experiment and target locations are all below ±1.3 nm. HODC, higher-order distortion correction; J-FIL, Jet and Flash Imprint Lithography.

**Figure 15:**
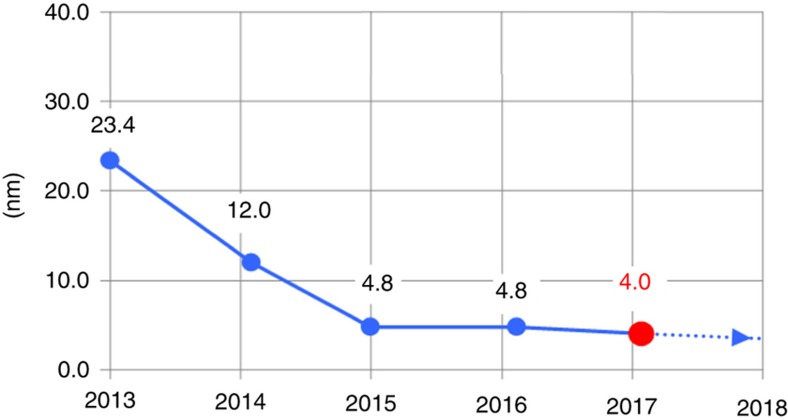
Overlay errors of 4 nm (mean+3 σ) was achieved when matching J-FIL to 193 nm wavelength immersion (193i) photolithography over a full 300 mm wafer with 84 stepper fields of size 26 mm×33 mm. J-FIL, Jet and Flash Imprint Lithography.

The J-FIL system includes several components that contribute towards achieving nanoscale overlay as discussed in section “The stepper system”. These components include the template MSCS, i-MAT, the precision *x*–*y*–θ air-bearing stage, and the HODC system. Figure 3 depicts the steps in the J-FIL process. In Step 3, the micro-scale template deformation causes high in-plane overlay errors making it very difficult to start the overlay process. Therefore, after the template has relaxed back to its nominally flat form in Step 4 of Figure 3, overlay correction is initiated. First, the i-MAT system provides eight independent alignment errors (*x* and *y* errors near the template corners). The i-MAT system operates over visible wavelengths at >500 Hz, and its signal quality is unaffected by UV exposure. Further, i-MAT does not block the UV exposure path, thereby providing *in situ* overlay error feedback at >500 Hz. These errors are converted into rigid body errors (*x*, *y*, θ), scale errors (independent magnification errors in *x* and *y*), and shape errors (orthogonality errors, and trapezoidal errors in *x* and *y*). The air-bearing stage is used to correct for the rigid body errors, and the MSCS corrects for the scale and shape errors. Errors that are higher order than the rigid body, scale, and shape errors cannot be detected in real time with the 8-channel i-MAT system. However, if such errors are systematic in nature and are present in each field, or repeat from wafer to wafer, they can be identified offline using a dense array of printed overlay marks within a field. Then, the HODC system can be used to correct for these systematic higher-order distortions in a feed-forward manner, as they cannot be corrected in real-time by the stage or the MSCS. Figure 14 shows the performance of the HODC while attempting to correct a known higher distortion error, the K11 “bow-shaped” error (reproduced with permission from Ref. 69). Figure 15 shows the recent improvement trend in overlay errors as measured over full 300 mm wafers with 84 stepper fields (including full and partial fields), while matching a J-FIL pattern to a previously lithographed 193i PL pattern (known as mix-and-match overlay—MMO). Distinct parasitic signatures associated with J-FIL and 193i PL need to be accounted for. The major improvement from year 2013 to 2015 has come from improvements in overlay on partial fields near the wafer edge. It should be noted that a single machine overlay (SMO), wherein two patterning layers were printed on the same J-FIL stepper, has yielded an overlay error of 2.1 nm (mean+3σ)^69^. The SMO and MMO results exceed Flash memory requirements in Table 1, and almost achieve those for DRAM^69,97^.

### Defect control

**Figure 16:**
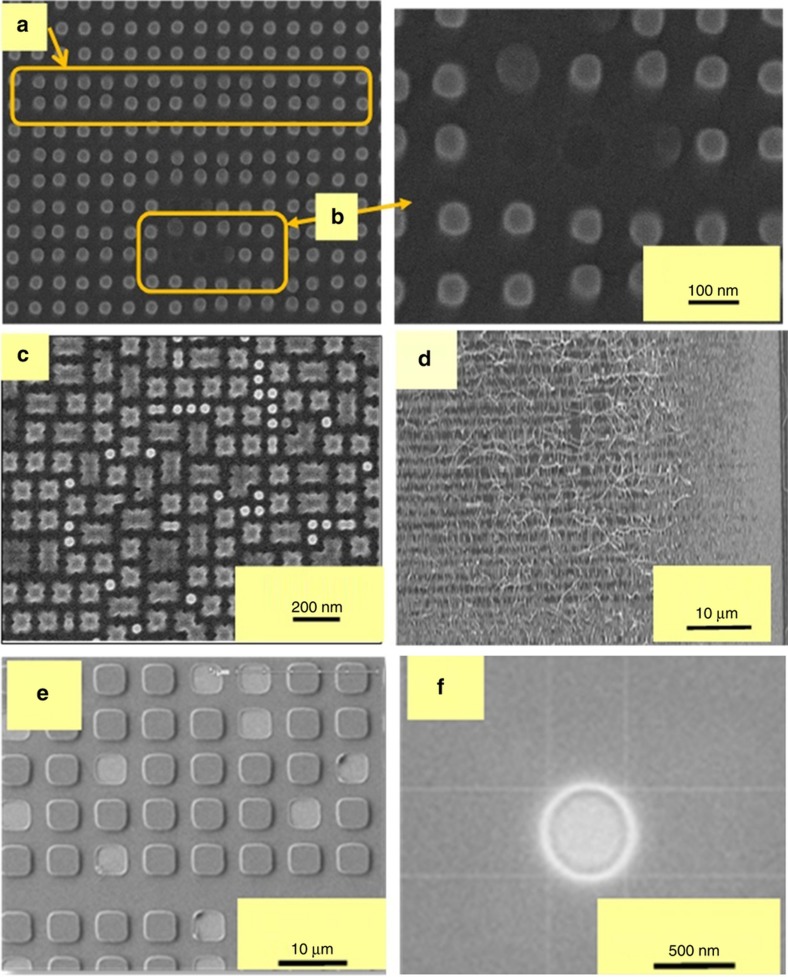
J-FIL solid phase defects (post UV cure, **a**–**d**); liquid phase (pre-UV cure, **e** and **f**). J-FIL, Jet and Flash Imprint Lithography; UV, ultraviolet.

In addition to particle-induced template repeaters (discussed in section “Particle contamination and template life”), non-repeating defects can occur in the liquid phase (before UV curing) or solid phase (after UV curing). Liquid phase defects include bubbles and micro- or nanoscale voids, while solid phase defects include separation induced shear, cohesive failure of imprint materials, and feature collapse defects during or after separation. The solutions to liquid phase and solid phase defects are well understood^98^ and are summarized here. Solid phase defects are illustrated in Figures 16a–d. Defect types A, B, and C are illustrated using resist nanopillars that are the least stable resist structure possible (resist holes being the most stable). Type A defects (local feature distortion) are caused by local shear stresses; type B (cohesive failure of resist) are caused by low resist strength or high aspect ratio pillars; type C defects (feature collapse) defects are caused by low resist modulus, high aspect ratio pillars, or very small spacing between pillars; and type D defects (large-scale shear) are caused by macroscale shear stresses induced by uncontrolled, high-speed delamination during the separation step (last step of Figure 3). A combination of enhanced resist materials, aspect ratios of <~2.5, and precision machine controls during the separation step have mitigated these types of defects. The liquid phase defects (Figures 16e and f) include incomplete filling defects (Figure 16e) and surface contamination voids (Figure 16f). Type E defects can be mitigated using precise fluid distribution combined with sufficient time for fluid filling (*t*_f_). The trade-off between fluid fill time and liquid phase defects, and similarly between separation time and solid phase defects imply that for any throughput data to be meaningful, the associated defect data has to be measured using advanced inspection tools that have the required resolution and adequate speed needed to provide large area statistics (for example, the KT 2915 series tools). Defect type F is known to be caused by airborne organic vapors that adsorb onto the adhesion layer (Figure 9) causing local dewetting spots.

The resist circumvents these spots leaving behind sub-micron-scale voids, which take the form shown in Figure 16f. These voids have been eliminated by avoiding exposure to airborne organics prior to entering the J-FIL stepper, and by ensuring that the J-FIL stepper is free from such contaminants.

**Figure 17:**
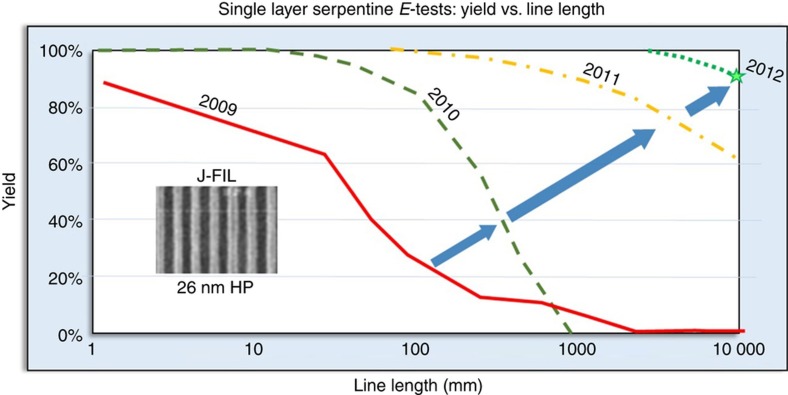
*E*-test yield results for 26 nm half-pitch serpentines.

An important milestone in the defect program is obtaining high yield in electrical tests (*E*-tests). *E*-tests represent a functional check of the patterning process and requires J-FIL to be integrated into the fab with other unit processes such as metallization and etch. The simplest *E*-tests involve fabrication and testing of single layer serpentines, while the ultimate *E*-test involves integrating J-FIL into one or more lithography steps in a memory product to investigate yield. These J-FIL-based *E*-tests require a collaboration with a leading-edge IC manufacturer. A collaboration with Toshiba between 2008 and 2012 at 26 nm half-pitch patterning resulted in the single layer serpentine yield data shown in Figure 17. A yield of >90% was targeted at 10-m-long serpentine lines as a milestone to validate J-FIL for memory production. This was achieved in 2012. The result achieved in 2012 was a culmination of a project involving substantially mitigating the particle contamination issues discussed in section “Particle contamination and template life”, and the defect types discussed in Figure 16. In this study, no further details such as opens *vs.* shorts, or the dominant defect types that cause yield loss was published. While defect densities have continually improved since 2012, Figure 17 is the only published J-FIL *E*-test data in the literature.

### Throughput

**Figure 18:**
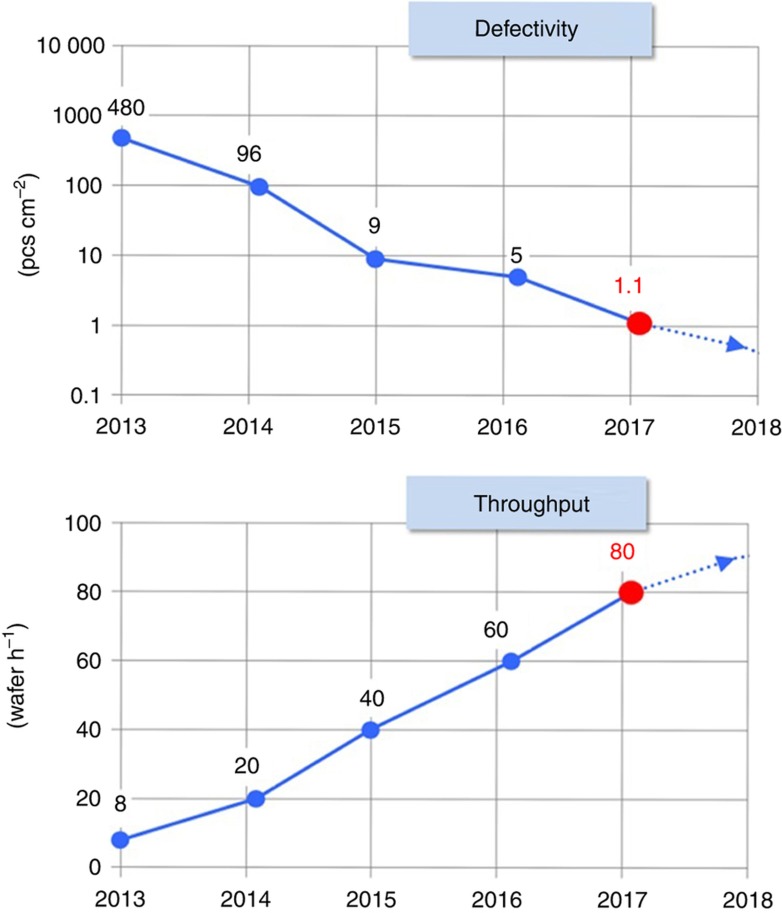
Optimal J-FIL process trend and acceptable defectivity for maximum throughput. J-FIL, Jet and Flash Imprint Lithography.

Template fluid filling is known to be the bottleneck in J-FIL as discussed in section “J-FIL stepper risks in semiconductor fabrication”. Complete fluid filling is achieved only when all the bubbles/voids in the template–fluid–substrate sandwich have disappeared. Overlay control takes place in parallel to template filling. Steps 3 and 4 in Figure 3 and further detailed in Figure 4 represent the throughput bottleneck. These steps take up ~65–70% of the throughput budget^69^. Steps 1 and 2 take up <20% of the throughput budget and more importantly depend on mature technologies such as high-speed *x*–*y* stages with micro-scale precision alignment relative to the template. Step 5, while very important for defects, only takes ~5% of the throughput budget. As discussed in section “Defect control”, increase in fluid filling time (*t*_f_) leads to a decrease in non-fill defects. Therefore, an optimal J-FIL process is set to run at the shortest *t*_f_ that leads to an acceptable defect density. As discussed in section “Semiconductor lithography requirements”, flash memory device yield requires defect density of ~1–10 defect cm^−2^, which is much higher than logic devices (see Table 1). This is because there exists a built-in redundancy in flash memory devices and this is one of the reasons it was chosen as the first target market (see section “Initial target market”). Figure 18 shows the defect densities being reduced to the low end of the 1–10 defects cm^−^^2^ while simultaneously achieving 80 WPH throughput. (The 80 WPH throughput was achieved in a four-station J-FIL stepper with each station running at 20 WPH.) This defect density is obtained using a KLA-Tencor 2915 Inspection tool and industry standard statistical protocols. This tool is capable of identifying defects for both 2x nm and 1x nm memory device patterning^99^. (Here 2x nm refers to 20–29 nm half-pitch, and 1x nm refers to ~15–19 nm half-pitch device design rules.) This represents the current state of the art for the optimal J-FIL process, wherein *t*_f_=1.1 s per field with the defect density approaching 1 defect cm^−2^. Various technical aspects that affect J-FIL fill time (*t*_f_) including improvements related to smaller and better-controlled inkjet drops, better wettability of drops on the wafer adhesion layer, template design rules for J-FIL, and machine control strategies are discussed in detail in the literature^88^.

## Concluding remarks

**Figure 19:**
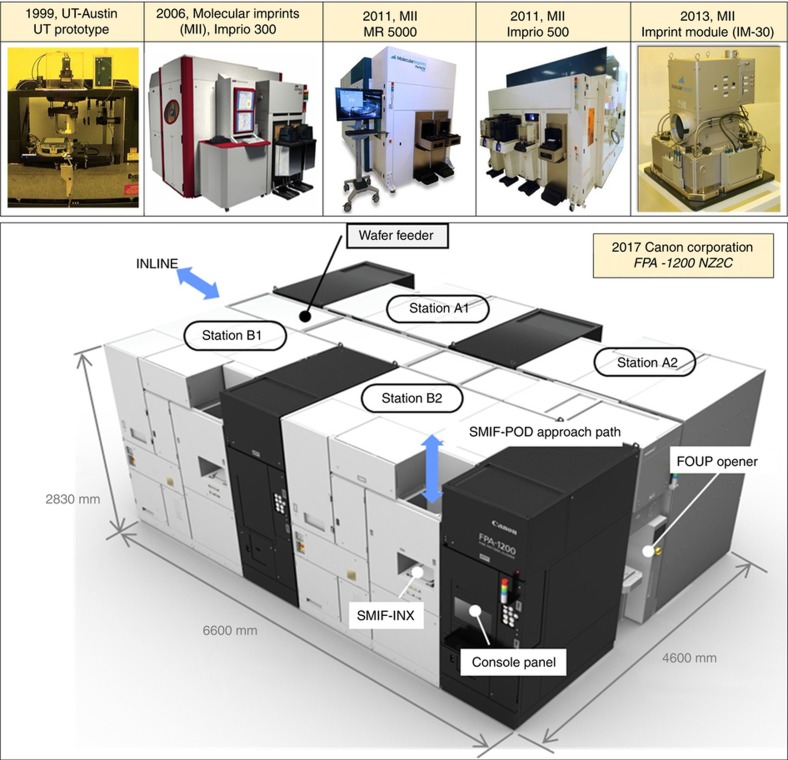
Major milestones of the J-FIL stepper system starting from the first UT-Austin prototype in 1999 to the four-station HVM tool deployed in 2017^100^. J-FIL, Jet and Flash Imprint Lithography.

This article describes the evolution of the J-FIL stepper technology, from university research to the recently deployed HVM product for fabrication of semiconductor ICs^100^. This represents the only NIL effort, and the only non-optical patterning technique, that has created an industrially viable lithography technology for advanced IC production. Figure 19 presents the key milestones in the evolution of the J-FIL stepper. The UT-Austin prototype developed in 1999 demonstrated the first use of inkjet (single nozzle) resist dispense in a nanoimprint stepper and verified the replication resolution below 50 nm over a 25 mm×25 mm stepper field. Imprio 300 incorporated a multinozzle (>100 nozzles) piezo inkjet, and deployed the first version of the MSCS (see Figure 8) to enable sub-20 nm MMO with PL. MR-5000 was the first template replicator developed and installed at mask manufacturer, DNP, to address the topic of template life (see section “Initial target market”). Imprio 500 was the first system to include two imprint stations, and improved various aspects of overlay, throughput and contamination control. IM-30 was the first IM (see section “HVM stepper development partnership” and Figure 5) developed by MII to enable a collaboration with Canon Corporation for development of production steppers. Finally, FPA-1200-NZ2C, a four-station HVM stepper for advanced memory production, was deployed by Canon in 2017 (Ref. 100). As discussed in an earlier section, this stepper has demonstrated: (i) ~0.0008 particles added per wafer processed through it (Figure 10), (ii) ~4 nm (mean+3σ) MMO (Figure 15), and (iii) 80WPH throughput at ~1.1 defects cm^−^^2^ (Figure 18).

A collaboration among the lithography ecosystem partners and memory manufacturers has led to deployment of stepper systems, lithographic materials, inkjets, and commercial templates, and to large area electrical yield data compatible with memory device manufacturing. The key remaining challenge for achieving the specifications set forth in Table 1 is template life of >1000 wafers. Systems that have demonstrated <0.001 added particles per wafer pass (PPWP) (Figure 10) are being made available to memory IC fabs where template life studies will be scaled up. Based on studies that have correlated PPWP and template life, it is expected that <0.001 PPWP will result in a template life of >1000 wafers by the end of this year^92^. Also, ongoing efforts by IC manufacturers involve developing device-specific process integration to deploy J-FIL in memory production. To extend J-FIL to production of logic devices, a further decrease in defect densities by at least two orders of magnitude is required, which is also being explored by Canon and its partners.

As discussed earlier, the only two lithographic technologies being considered for IC production beyond 193i PL to pattern 20 nm half-pitch structures with arbitrary complexity are J-FIL and EUV. Section “EUV lithography” described that EUVL has to address the coupling between resolution/LER and throughput caused by fundamental shot noise limitations. This requires continued increase in source power which causes fundamental challenges for the creation of reliable pellicles and optical systems. Also, a number of system engineering challenges have to be addressed for roadmap extendibility in EUVL. The inherent resolution advantage of J-FIL—its demonstrated ability to pattern sub-5 nm resist structures and complicated patterns—appears to make the J-FIL technology roadmap more readily extendable. The technology has the potential be the same at higher resolutions, requiring incremental improvements in overlay and defect management. To address this extendability, Canon recently presented a NIL roadmap with planned advances in resolution, overlay, throughput, defects, and template life out to year 2021 (Ref. 69)^.^

Leading-edge semiconductor lithography has some of the most aggressive technology requirements. Commercialization of this challenging nanomanufacturing technology has resulted in important observations that may be more broadly applicable to researchers involved in other nanomanufacturing technologies:

- *Large strategic market*: Nanolithography is a key driver in the important business of semiconductor ICs which has justified significant investments in the research-and development phases of this disruptive technology.
- E*xtendable technology*: The fact that NIL’s inherent resolution is well below 5 nm half-pitch makes it likely that this technology would be applicable well beyond its initial introduction at around 20 nm half-pitch structures.
- *Early adopters*: The customer partnerships in the advanced memory fabrication sector have allowed for a careful documentation of the minimally acceptable specifications that can create commercial value.
- *Leveraging existing infrastructure*: Partnering with well-established IC fab ecosystem suppliers that bring in complementary capabilities has helped in sharing of the technical risk and the reward of deploying this new technology.
- *Shared technology roadmap*: A carefully planned technology development roadmap among all the partners—that established intermediate milestones to systematically overcome the major technical risks—has enabled investments from both the public and the private sectors. The execution of this technology roadmap has, for example, led to a HVM stepper system (Figure 19) that was recently deployed to a memory fab by Canon^100^.
- *Trusted system-level models*: An important area of future research needs to be the creation of trusted multi-scale models in the area of nanomanufacturing. An inefficient, trial-and-error experimental approach, such as the one described in this paper, still the norm during the development of new nanomanufacturing technologies. The primary reason for this is the absence of comprehensive and reliable system-level models that possess nanoscale precision over large scales.

## Figures and Tables

**Table 1:** Representative semiconductor IC fabrication requirements. Actual requirements can vary somewhat for specific devices and device layers. These are approximate estimates for advanced memory, both Flash and DRAM.^a^

No.	Lithography specification description	Specification
1.	Substrate size/type	300 mm diameter silicon
2.	Patterning area per process step (field size)	26 mm×33 mm
3.	Substrate flatness over a field (peak-to-valley)	<25 nm, spatial periods of >5 mm
4.	CD (min. feature size and half-pitch)	<20 nm half-pitch
5.	Line-edge roughness (3σ)	<2 nm
6.	CDU (3σ)	<2 nm
7.	Wafer defect density, logic *vs.* memory (relevant size >0.5×CD)	<0.01/cm^2^ *vs. <*1–10/cm^2^ (defect size >10 nm)
8.	Photomask usage *vs.* NIL template life^b^	>5000 wafers *vs.* >1000 wafers with replication^b^
9.	Alignment (*A*) and overlay (*O*) errors (mean+3σ)	Flash/DRAM: *A*<3 nm/2.5 nm; *O*<5 nm/3.5 nm
10.	Pattern layout, complexity	Cartesian, arbitrary patterns
11.	Pattern density variations	High
12.	Printing of partial fields over the wafer edge printing	Yes
13.	Throughput for acceptable equipment cost^b^	>5 WPH/US$1M of equipment cost

Abbreviations: CD, critical dimension; CDU, CD uniformity; IC, integrated circuit; NIL, nanoimprint lithography; WPH, wafers per hour.

a This table defines critical dimensions based on half-pitch of the finest structures for memory devices; logic-device definitions of lithographic nodes have become increasingly confusing over the past 15 years due to half-pitch of metal one layer diverging from the gate length. For example, Ref. 1 points out that Intel’s 65 nm node process in 2005 had a gate length of 32 nm and the half-pitch of the Metal 1 of 105 nm.

b Template life/cost and tool throughput/cost represent the dominant factors in NIL cost-of-ownership, other costs include process materials and gases. Template replication is a key complement to template life, see section “Resist and inkjet development strategy”.
